# Functional dissection of phytochrome A in plants

**DOI:** 10.3389/fpls.2024.1340260

**Published:** 2024-01-26

**Authors:** Yuqi Lei, Qi Ma, Yihao Zhang, Jilian Li, Xinzhu Ning, Yichen Wang, Xiaoyang Ge, Hang Zhao, Hai Lin

**Affiliations:** ^1^ Cotton Research Institute, Xinjiang Academy of Agricultural and Reclamation Science, Shihezi, China; ^2^ National Key Laboratory of Cotton Bio-breeding and Integrated Utilization, Institute of Cotton Research, Chinese Academy of Agricultural, Anyang, China; ^3^ Aulin College, Northeast Forestry University, Harbin, China; ^4^ College of Life Sciences, Qufu Normal University, Qufu, China

**Keywords:** phytochrome A, far-red light signaling, flowering time, shade avoidance, light signaling

## Abstract

Plants lack behavioral responses to avoid dramatic environmental changes associated with the annual seasons. For survival, they have evolved complex sensory systems to sense fluctuations in light and optimize their architecture in response to changes in these cues. Phytochrome A (phyA) was initially identified as a photoreceptor that senses far-red light signals. It was then identified as playing a central role in promoting hypocotyl growth, fiber development, and flowering time in a variety of plants including Arabidopsis, rice, soybean and cotton. Under dark conditions, phyA is present in the cytoplasm in the physiologically inactive (Pr) form. Far-red light signals induce the transformation of Pr into the physiologically active (Pfr) form, after which Pfr-phyA is recognized by FAR-RED ELONGATED HYPOCOTYL 1 (FHY1) and FHY1-LIKE (FHL) and translocated to the nucleus, initiating a series of signaling cascades. The current review comprehensively summarizes recent advances in understanding the function of phyA in plants, including phyA-mediated shade avoidance and flowering time. Remaining issues and possible directions for future research on phyA are also discussed.

## 1 Introduction

As sessile organisms, plants encounter a range of adverse environmental influences throughout their entire life cycle. To adapt to these unfavorable conditions, plants exhibit a noteworthy form of adaptability (Bellard et al., 2012; Legris et al., 2017). For example, over the course of their lengthy evolutionary journey, plants have gradually evolved distinct light-sensing systems, referred to as photoreceptors or “eyes” of plants (Zhao and Bao, 2021; Fang et al., 2022). These photoreceptors grant plants the ability to perceive environmental light characteristics, including light intensity, photoperiod, and a broad spectrum of light wavelengths that elude human perception. They translate these light signals into internal physiological cues, subsequently governing their growth and development in response to fluctuations in light conditions (Cheng et al., 2021a; Fichman et al., 2023; Yuan et al., 2023).

One of the most pivotal photoreceptors in plants for light perception is the phytochromes, which can detect signals in the red light range (wavelength between 600 and 700 nm) and the far-red light range (wavelength between 700 and 760 nm) (Wang and Deng, 2003; Wang et al., 2022a). As early as the 1950s, botanists observed that plant seeds exhibited higher germination rates under red light, while germination rates were notably lower under far-red light. The observation prompted the hypothesis that plants contain a pigment capable of absorbing red or far-red light, thereby reversibly influencing plant development. Scientists subsequently isolated the photoreceptor responsible for sensing red and far-red light, termed phytochrome. Depending on the wavelength of light they perceive, phytochromes can be categorized into two types: those absorbing far-red light (Pfr, physiologically active) and those absorbing red light (Pr, physiologically inactive). These two forms have the ability to be interconverted (Paul and Khurana, 2008).

Researches have indicated that in the majority of dicotyledonous plants, with *Arabidopsis thaliana* serving as a representative example, phytochromes are encoded by five gene families, specifically *PHYA* to *PHYE* (Clack et al., 1994; Abdurakhmonov et al., 2010). In monocotyledonous plants, phytochromes are encoded by three genes, *PHYA*, *PHYB*, and *PHYC*. This divergence may represent a strategy employed by plants during their domestication process to adapt to their surroundings. phyA, a crucial type of phytochromes common to both monocots and dicots, plays a primary role in the transduction of far-red light signals (**Table 1**) (Casal et al., 2014; Choi et al., 2023; Zhang et al., 2023a). In the dark, phyA protein is synthesized and exists in the cytoplasm in the Pr form. Upon detecting far-red light signals, the conformation of phyA undergoes a change, transforming it into the active Pfr form (Kami et al., 2010; Hughes, 2013). FHY1 and FHL can function as transporters, facilitating the nuclear import of phyA and mediating its entry into the nucleus through interaction with Pfr-phyA (Hiltbrunner et al., 2005; Hiltbrunner et al., 2006). Once phyA enters the nucleus, it interacts with a variety of factors that regulate light signals, including phytochrome interacting factors (PIFs), MYB30, and SUPPRESSOR OF PHYTOCHROME A 1 (SPA1) (**Table 1**), initiating a sequence of signal cascades (Lorrain et al., 2009; Seaton et al., 2018; Yan et al., 2020; Yuan et al., 2023). This subsequently leads to alterations in the expression of light-responsive genes, which, in turn, govern various biological processes like seed germination, de-etiolation, hypocotyl growth, and flowering (Cerdan and Chory, 2003; Barros-Galvao et al., 2020; Zeng et al., 2022).

**Table 1:** phyA-interacting proteins in regulating flowering time and hypocotyl growth.

Protein Interaction	Experimental Technology	Molecular Function	Biological Function	Ref.
AtphyA-PIF3	Y2H, Pull-down	Regulate PIF3 by phosphorylation	Negatively regulate photomorphogenic development	(Ni et al., 1998)
AtphyA-COP1	BiFc, Pull-down	COP1 acts as an E3 ligase to ubiquitinate phyA	Negatively regulate photomorphogenic development	(Seo et al., 2004)
AtphyA-PIF1	Pull-down	Regulate PIF1 by phosphorylation	Negatively regulate photomorphogenic development	(Oh et al., 2004)
AtphyA- AtIAA1/IAA3/IAA7/IAA17	LCI, Pull-down, BiFC	Prevent degradation of IAA17	Inhibit hypocotyl elongation	(Yang et al., 2018)
AtphyA-AtMYB30	Pull-down, LCI, Co-IP	Stabilize MYB30 in the light	Negatively regulate photomorphogenic development	(Yan et al., 2020)
AtphyA-AtTIC	Co-IP, BiFC	Repress *PHYA* transcription	Negative regulator of light -inhibited hypocotyl growth	(Cheng et al., 2021b)
AtphyA-AtFIN219	Pull-down, BiFC, Co-IP	Suppress phyA activity by regulate phyA subcellular localization	Inhibit hypocotyl elongation	(Jiang et al., 2023)
AtphyA-SPA1	Y2H, BiFC	Mediate FR-induced disassociation of COP1 from SPA1	Accelerate flowering	(He et al., 2018)
AtphyA-FHY1	Y2H, Pull down	Light-regulated phytochrome nuclear accumulation.		(Hiltbrunner et al., 2005)
AtphyA-AtTZP	Y2H, LCI, Pull down, Co-IP	Regulates phyA phosphorylation in the Nucleus in FR Light		(Liu et al., 2021; Sang et al., 2021)
ZmphyA1/2-PIFs	Y2H,LCI			(Cao et al., 2023)
OsphyA-Ghd7	Y2H, Pull-down, BIFC, Co-IP	Stabilize Ghd7 by copeting with OsGI for binding to Ghd7	Delay flowering	(Zheng et al., 2019)
GmphyA2/3-GmLUC1/2 GmphyA2/3-E1	Y2H, Co-IP Y2H, Pull-down, Co-IP,	Degrade LUX Stabilize E1	Delay flowering Delay flowering	(Lin et al., 2022) (Lin et al., 2022)
BdphyA-BdPIL1/BdPIL3	Y2H, Pull-down	Prevent BdPIL1/PIL3 binding to promoters of downstream genes	Negatively regulate floral induction	(Hoang et al., 2021)

## 2 Regulation of plant flowering time by phyA

Plants can perceive seasonal changes in photoperiod through phytochromes, ensuring they flower at the appropriate time (Guo et al., 1998; Cerdan and Chory, 2003; Zhao et al., 2023a). In the long-day plant *Arabidopsis thaliana*, it has been observed that white light supplemented with far-red light is more effective at promoting flowering than white light alone, highlighting the significant role of far-red light in triggering flowering in Arabidopsis (Whitelam et al., 1993; Johnson et al., 1994). In-depth studies have revealed that phyA and phyB can temporally and dynamically regulate the protein levels of CONSTANT (CO), a B-box zinc finger protein, ensuring that plants recognize specific photoperiodic environments and thus precisely regulate flowering. In the morning, active phyB promotes CO protein degradation, leading it to lower levels of CO protein (Hajdu et al., 2015; Zhang et al., 2023b). In the afternoon phyA stabilizes the CO protein through the inhibition of the ubiquitin ligase activity of CULLIN 4 (CUL4)- DNA DAMAGE-BINDING PROTEIN 1 (DDB1) ^COP1/SPA^. Consequently, this enhances CO’s transcriptional activation of the flowering-inducing gene *FLOWERING LOCUS T* (*FT)*, ultimately promoting flowering in Arabidopsis (Yanovsky and Kay, 2002; Saijo et al., 2008; Lau and Deng, 2012).Taken together, Arabidopsis phyA and phyB have opposite functions in regulating flowering, thereby recognizing specific photoperiodic environments and precisely regulating flowering.

Phytochromes not only mediate flowering in *Arabidopsis* through the CO-FT pathway but also regulate flowering and growth via PIF regulation. The Pfr-phyB form interacts with PIF4, promoting its degradation and inhibiting plant flowering and growth. Additionally, Pfr-phyB physically interacts with and inhibits COP1, leading to ELONGATED HYPOCOTYL 5 (HY5) accumulation and subsequent inhibition of PIF4 transcripts. Importantly, phyB can complex with EARLY FLOWERING 3 (ELF3) and the E3 ubiquitin ligase HIGH EXPRESSION OF OSMOTICALLY RESPONSIVE GENES 1 (HOS1), aiding ELF3 and HOS1 in preventing PIF4 binding to target genes, including *PIL1* and *Auxin/indole-3-acetic acid* (*IAA19)*, thus blocking the PIF4 signaling pathway. The impact of this mechanism on the flowering process warrants further investigation. In contrast to phyB, phyA interacts with PIF3, aiding in PIF3 degradation and positively regulating seedling de-yellowing. While phyA’s role in Arabidopsis flowering regulation via PIF3 remains unreported, its interaction with BdPIL1/BdPIL3 in Brachypodium distachyon suggests a physiological function in promoting inflorescence induction (Ni et al., 1998; Kim et al., 2003; Hoang et al., 2021).

**Figure 1:**
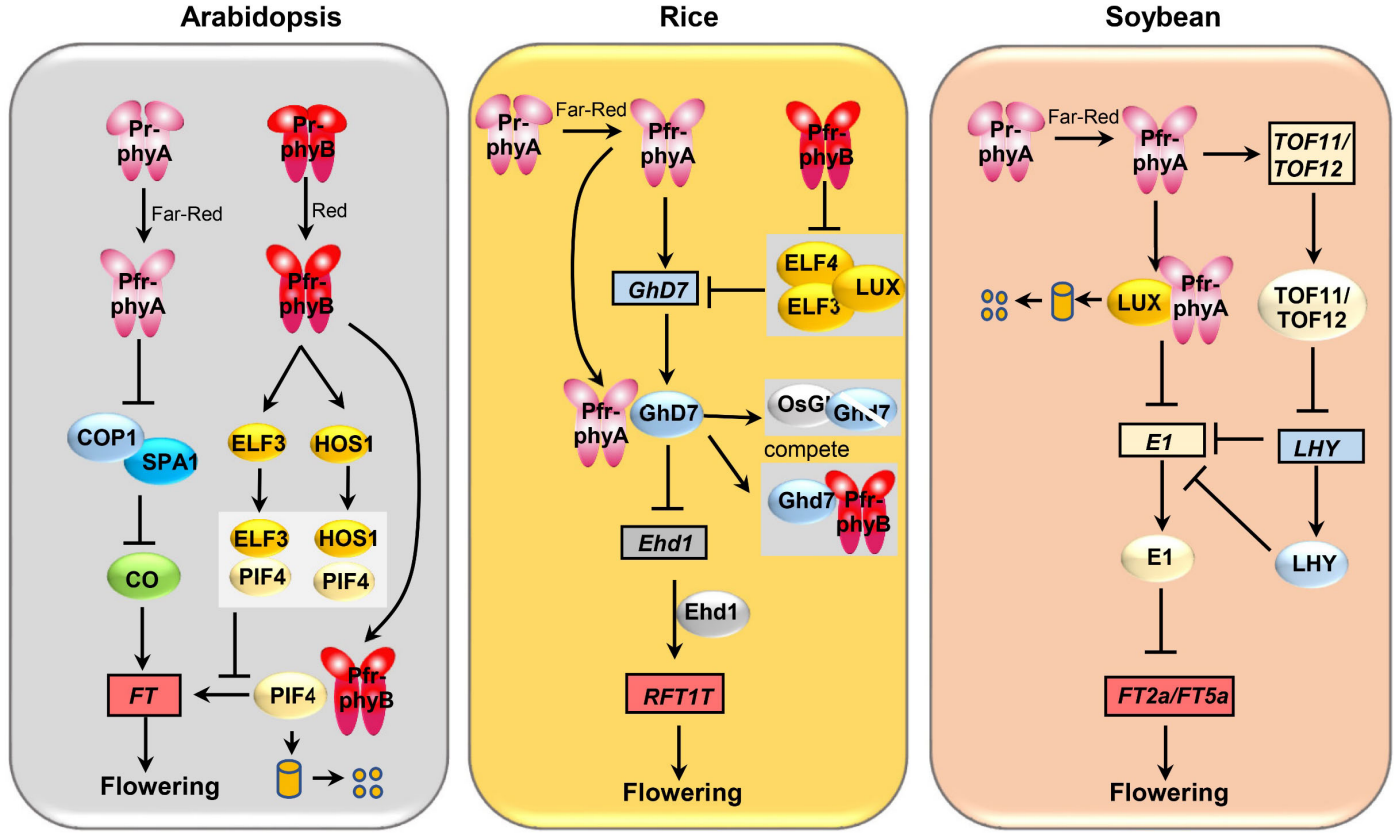
Comparison of the genetic pathways underlying phyA-mediated flowering regulation in soybean, rice, and Arabidopsis. Boxes represent genes and ovals represent proteins.

In addition to their role in regulating flowering in long-day Arabidopsis, phytochromes also play a part in controlling flowering in short-day plants such as rice and soybean (**Figure 1**). However, the specific functions and molecular mechanisms governing flowering regulation vary among different crops. Research has revealed that under natural long-day conditions, single mutations in *PHYB* and *PHYC* result in a moderately earlier flowering phenotype in rice, whereas the *PHYA* single mutation shows no significant difference in flowering time compared to the wild type (Takano et al., 2005; Bae and Choi, 2008). Nevertheless, double mutations in *PHYAPHYB* and *PHYA PHYC* lead to significantly earlier flowering compared to both the wild type and the single gene mutants *phyB* and *PHYCphyC*, suggesting phyA, phyB, and phyC exhibit functional redundancy in regulating rice flowering, with phyA potentially not being the primary regulator of flowering (Takano et al., 2005). Mechanistically, rice phytochromes can regulate the transcription and protein stability of Grain number, plant height and heading date 7 (Ghd7) through multiple pathways, consequently affecting flowering time. Firstly, under long-day conditions, phyA can induce the expression of the core flowering repressor, *Ghd7*, in rice, which, in turn, strengthens Ghd7’s ability to inhibit the Early heading date 1(Ehd1)-RICE FLOWERING LOCUS T 1 (RFT1) pathway, thus negatively regulating flowering time (Osugi et al., 2011). phyB, by inhibiting the protein activity of the evening complex component EARLY FLOWERING 3 (ELF3) (Zhao et al., 2021), relieves evening complex’s transcriptional inhibition of *Ghd7*, causing a delay in flowering (Andrade et al., 2022). It’s worth noting that phyA does not regulate the protein activity of OsELF3, highlighting the specificity of phyA and phyB in the regulation of *Ghd7* transcription levels. Remarkably, aside from its role in inducing *Ghd7* at the transcriptional level, phyA and phyB can also compete with *Oryza sativa* GIGANTEA (OsGI) proteins to interact with Ghd7 proteins. This competition rescues Ghd7 from degradation by OsGI, thereby stabilizing Ghd7 proteins and leading to delayed flowering (Zheng et al., 2019). In summary, phytochromes regulate rice flowering through various pathways, and exploring whether these pathways exhibit functional conservation across different plant species represents a valuable avenue for further research.

In contrast to the dominant role of phyB in regulating flowering in rice, phyA has been established as the primary gene responsible for photoperiod-induced flowering in the short-day crop soybean. Studies have shown that under long-day conditions, *phyA2phyA3* exhibit significantly earlier flowering phenotypes compared to the wild type, whereas *phyB1phyB2* do not exhibit a significant difference in flowering time compared to the wild type (Lin et al., 2022). This observation suggests that phyA plays a more crucial role than phyB in soybean flowering. Interestingly, unlike other plants, soybean lacks CO protein or homologs that are regulated by phytochromes, and instead has a specific flowering repressor, E1, whose transcription is inhibited by LUX ARRYTHMO (LUX) (Bu et al., 2021). Further investigations have revealed that phyA2 and phyA3 can interact with LUX, leading to the degradation of GmLUX. This, in turn, releases GmLUX’s transcriptional inhibition of the soybean-specific flowering repressor E1, resulting in the accumulation of E1. The increased E1 levels inhibit the transcription of the flowering genes *FT2a* and *FT5a*, thereby delaying flowering in soybean (Lin et al., 2022; Zhao et al., 2023b). Intriguingly, phyA2 and phyA3 can also directly interact with E1, enhancing the stability of E1 and further negatively regulating soybean flowering (Lin et al., 2022). In addition, phyA2 and phyA3 were able to induce the expression of *Time of Flowering 11* (*TOF11*) and *TOF12*, two PRR3 homeologs that inhibit the expression of LHYs, and in turn releases the inhibitory effect of LATE ELONGATED HUPOCOTYL (LHYs) on E1, ultimately resulting in the inhibition of flowering (Lu et al., 2020). Recent research has additionally demonstrated that, aside from its involvement in the photoperiod, phyA also participates in temperature-mediated flowering in soybean. An increase in temperature from 25°C to 30°C induces the expression of *FT2a* and *FT5a* and accelerates flowering in soybean. However, a further increase in temperature from 30°C to 35°C activates the phyA-E1 pathway, causing a delay in flowering (Tang et al., 2023).

As the functions and significance of phyA continue to unfold, its role in flowering regulation has been documented in various species. In Medicago, MtphyA is known to induce the transcription of genes like *MtE1L* and *MtFT*, thereby promoting flowering (Jaudal et al., 2020). In leafy mustard (*Brassica juncea*), PHYTOCHROME A SIGNAL TRANSDUCTION1 (PAT1), a positive regulator of phyA signaling, interacts with CONSTANS-LIKE 13 (COL13) to negatively regulate flowering (Muntha et al., 2019). In cotton, *PHYA* RNAi-silenced plants display characteristics such as early maturity, longer fibers, and improved fiber quality (Abdurakhmonov et al., 2014). This suggests the potential for phytochromes to aggregate a range of desirable agronomic traits, offering a novel approach to breeding early-maturing, high-yielding, and high-quality upland cotton varieties (Abdurakhmonov et al., 2014). Nevertheless, the precise mechanism through which cotton phyA regulates plant flowering is still an area that warrants further exploration. In summary, phyA plays a role in regulating flowering time in various crops, with its specific flowering regulatory functions and molecular mechanisms varying among different species. In the future, meaningful research avenues could involve studying the potential of regulation of phytochrome activity or the modification of key components and sites within the phytochrome signaling pathways in molecular breeding. This approach holds promise for obtaining desirable traits in crops.

## 3 Regulation of plant shade avoidance by phyA

Plants lack mobility, and the intensity of light in their natural environment often decreases due to the shading effect of neighboring plant canopies, leading to a reduction in the red to far-red light ratio (R:FR). When phytochromes perceive this change in light intensity, it triggers a series of adaptive responses, also known as shade avoidance syndrome (Liu et al., 2019; Zhang et al., 2019; Sun et al., 2020). Shade avoidance is evident through the elongation of the hypocotyl, stem, and petioles, allowing the plant to compete for more sources of light with surrounding plants (Fernandez-Milmanda and Ballare, 2021).

Research has demonstrated that phyA and phyB play antagonistic roles in the plant’s response to shade avoidance. In white light conditions, phyB suppresses hypocotyl elongation by directly inhibiting the transcription of cell elongation-related genes. When the R:FR ratio decreases in mild shading conditions, phyA primarily accumulates in the cytoplasm without actively functioning, whereas phyB becomes inactive, thereby relieving the transcriptional inhibition of elongation-related genes like PIF7, and resulting in hypocotyl elongation (Leivar and Monte, 2014; Zhang et al., 2019). However, when the R/FR ratio is very low, mimicking deep canopy shade, phyA is activated and transported to the cell nucleus. In molecular terms, phyA serves as a positive regulator of HY5, which itself is a key promoter of photomorphogenesis. This regulation by phyA effectively mitigates the exaggerated hypocotyl elongation typically triggered by the inactivation of phyB, thereby contributing to the reduction of the plant’s energy expenditure. Intriguingly, the phyB-HY5 signaling axis can be augmented by the TANDEM AINC-FINGER/PLUS3 (TZP) protein. TZP, functioning as an antagonist of hypocotyl elongation, is noted for its mutant forms displaying elongated hypocotyls under FR conditions. Further investigations have elucidated that TZP interacts with far-red light signaling through dual mechanisms. Initially, TZP has been shown to engage in direct interaction with phyA, leading to its phosphorylation; this post-translational modification of phyA consequently amplifies the phyA-HY5 signaling pathway. Additionally, TZP and HY5 are found to competitively bind to COP1, an interaction that enhances the stability of the HY5 protein and further suppresses hypocotyl elongation (Kaiserli et al., 2015; Zhang et al., 2018; Li et al., 2022). In addition to the phyB-HY5 pathway, phyA competes with the auxin/indole-3-acetic acid (AUX/IAA) complex through competitive interactions with the auxin receptor TIR (Transport Inhibitor Response), preventing the degradation of AUX/IAA and weakening auxin signaling (Salehin et al., 2015; Yang et al., 2018). Ultimately, this leads to a reduced shade avoidance response, preventing excessive seedling elongation. Recent research indicates that phyA can also ameliorate the detrimental effects of deep shade on plant growth by enhancing the expression of core components of the circadian clock, including TIMING OF CAB EXPRESSION1 (TOC1), PSEUDO-RESPONSE REGULATOR 7 (PRR7), ELF3, and ELF4 (Favero et al., 2021). Intriguingly, recent studies reveal that the protein abundance of phyA is negatively regulated by the circadian clock regulator TIME FOR COFFEE (TIC). TIC recruits TOPLESS (TPL) along with other transcription factors to bind to the promoter of the *PHYA* gene, effectively suppressing the transcriptional expression of *PHYA* at pre-dawn. Concurrently, TIC binds directly to phyA in the cell nucleus, facilitating the hydrolysis of phyA (Wang et al., 2022b). These findings imply that light signals and the biological clock may dynamically regulate plant development in response to environmental stress. Considering that dense cultivation is one of the effective ways for increasing crop yield per unit area, but dense cultivation can result in mutual shading among plants, triggering a cascade of shade-avoidance responses and ultimately reducing plant yield. In the future, investigating how to utilize the regulatory genes or signaling pathways mentioned above to enhance yield without compromising plant productivity will be a meaningful avenue of research.

**Figure 2:**
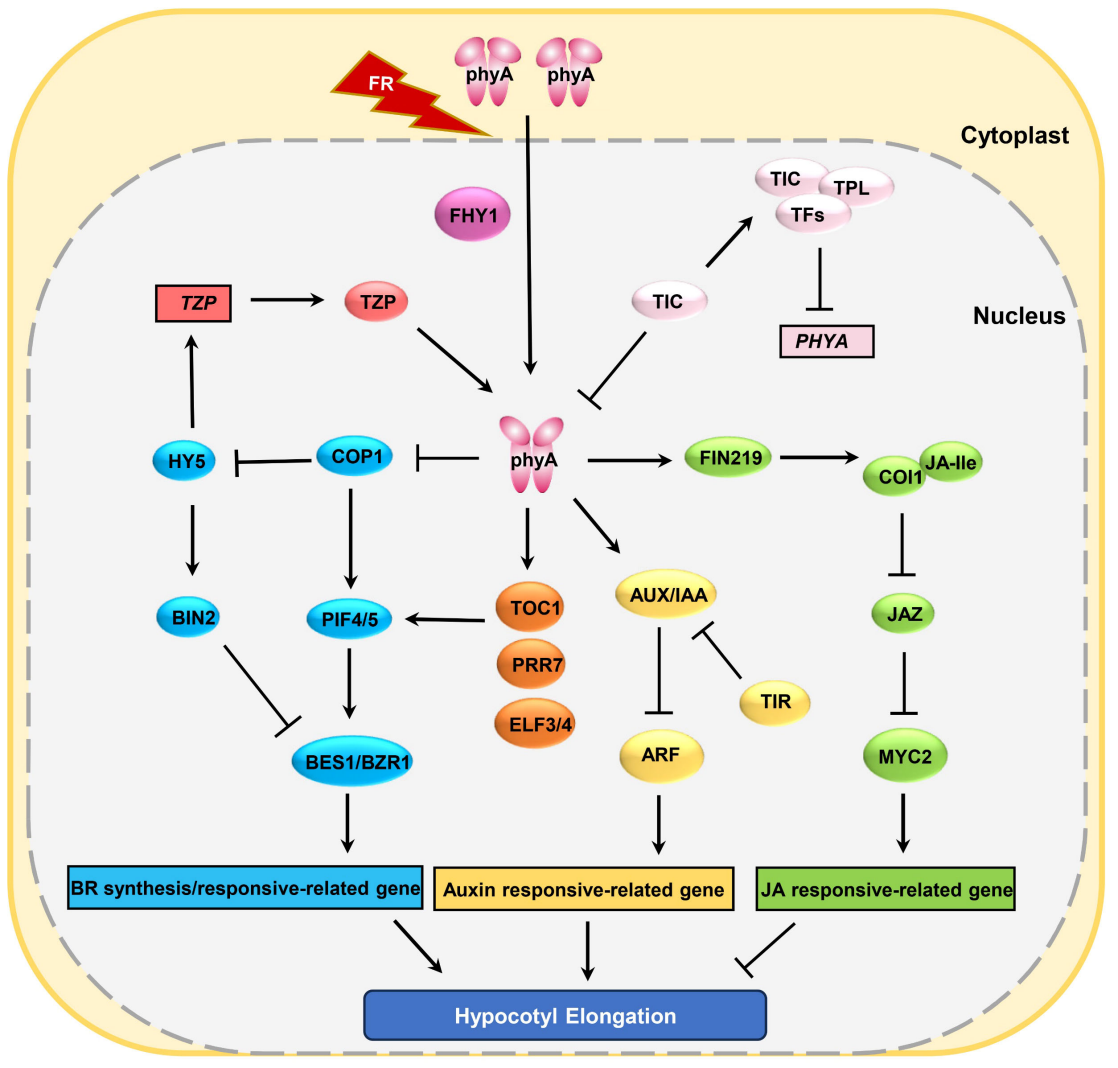
phyA regulates plant hypocotyl growth through multiple pathways. Boxes represent genes and ovals represent proteins.

In addition to the above-mentioned growth hormone signals, phyA is also involved in plant hypocotyl elongation mediated by various plant hormones such as jasmonic acid (JA) and brassinosteroid (BR) (**Figure 2**). Studies show that phyA inhibits hypocotyl elongation by suppressing the BR signaling pathway (Song et al., 2020; Zhao et al., 2022). By comparing the sensitivity of *phyA* to brassinazole (BRZ, a BR biosynthesis inhibitor) under mild and deep shade conditions, it was observed that *phyA* displays greater sensitivity to BRZ under deep shade (Song et al., 2020). This suggests that phyA may be operated by inhibiting the BR pathway in shaded conditions. Further research revealed that, after prolonged shade treatment, active phyA can reduce CONSTITUTIVELY PHOTOMORPHOGENIC 1 (COP1) protein accumulation in the nucleus leading to the decreased accumulation of COP1 downstream proteins like PIF4 and PIF5, and the increased level of COP1 downstream proteins ELONGATED HYPOCOTYL 5 (HY5), an inducer of *BRASSINOSTEROID-INSENSITIVE2* (*BIN2*), subsequently inhibiting the expression of BES1/BZR1 and BR biosynthesis-related genes (**Figure 2**), and suppressing hypocotyl elongation (Li et al., 2020; Song et al., 2020). Besides BR, JA, a hormone responsible for regulating plant stress resistance, also modulates hypocotyl growth. Research suggests that phyA primarily regulates JA biosynthesis and signal transduction to suppress hypocotyl elongation. phyA positively regulates the protein abundance of JASMONATE RESISTANCE 1 (JAR1) and FAR-RED INSENSITIVE 219 (FIN219), a JA-conjugating enzyme for the generation of an active JA-isoleucine (JA-Ile), promoting the production of JA-Ile. JA-Ile binds and activates COI1, resulting in the ubiquitination and degradation of JAZ transcriptional repressors. This, in turn, releases the MYC2 transcription factor, suppressing hypocotyl elongation (**Figure 2**) (Robson et al., 2010; Hsieh and Okamoto, 2014; Jiang et al., 2023).

In summary, phyA can integrate light signals, the circadian clock, and hormone signals to regulate plant growth. Moving forward, the identification of molecular targets for desirable agronomic traits in molecular breeding, as well as the development of novel strategies to improve crop yield and quality by various R/FR ratios or alterations in light quality, along with a comprehensive understanding of the interplay between phytochromes and the key components in their signaling pathway, holds significant importance.

## 4 Conclusion and future perspectives

Since the discovery of photoreceptors in the 1950s, researchers have gradually unveiled their expression patterns and optical properties. phyA, a phytochrome in plants capable of sensing far-red light signals, has its signaling pathways in plant growth, flowering, seed development, and other aspects elucidated (Zhou et al., 2018; Lin et al., 2022; Lee et al., 2023). The integration of these signaling pathways has bolstered the robustness of the regulatory network mediated by phyA. Despite significant progress in phyA research, there remain several unresolved issues and challenging tasks.

First and foremost, current research on phyA-mediated light signals in plant growth and flowering predominantly focuses on model plants such as Arabidopsis and rice. There is a scarcity of studies on economic crops and horticultural plants. In the future, efficient crop genetic transformation systems can be employed to investigate the function of phytochromes in crops. Molecular biology techniques, such as gene editing, can be harnessed to modify the signaling pathways of phytochromes, thus enhancing crop performance and yielding new crop varieties with outstanding agronomic traits, including stress resistance and high yield. Secondly, different light qualities exert varying effects on plant growth, development, metabolites, and stress resistance (Lin, 2000; Mao et al., 2020; Spaninks and Offringa, 2023). However, natural sunlight comprises a broad spectrum, making it challenging to isolate single-color spectra for practical use. In the future, plant factories can be established to provide plants with optimal light quality or design the ideal combination of spectra to guide the breeding of excellent crop varieties. Thirdly, ambient temperature, a pivotal environmental variable, exerts a significant influence on both the timing of flowering and hypocotyl growth in plants. Recent research has highlighted the role of phyA in the temperature-dependent regulation of flowering in soybean (Tang et al., 2023). This opens an intriguing avenue for future research: investigating whether the phyA-mediated temperature signaling mechanism observed in soybean is applicable to other crop species, and exploring potential strategies to harness this pathway for agricultural enhancement. Fourthly, both phyA and phyB interact with SWI2/SNF2-Related 1 (SWR1) complex subunits SWC6 and ARP6 to promote H2A.Z deposition at HY5 target genes and regulate HY5 target gene expression (Wei et al., 2021; Chen et al., 2023). It would be very interesting to delve into how phyA regulates flowering and plant growth by affecting chromatin conformation at the epigenetic level in different crops. Finally, it is crucial to recognize that natural environments are characterized by dynamic fluctuations in both light and temperature, a scenario that markedly contrasts with the static light or temperature conditions typically used in laboratory studies. Therefore, an in-depth exploration into how phyA integrates these fluctuating light and temperature signals to orchestrate plant flowering and growth processes is not only scientifically compelling but also holds considerable practical relevance.

## Author contributions

YL: Resources, Writing – original draft. QM: Investigation, Resources, Writing – review & editing. YZ: Writing – review & editing. JL: Funding acquisition, Writing – review & editing. XN: Resources, Validation, Writing – review & editing. YW: Writing – review & editing. XG: Writing – original draft, Writing – review & editing. HZ: Writing – original draft. HL: Resources, Writing – original draft, Writing – review & editing.
